# Supplementary material for: Galanin System in the Human Bile Duct and Perihilar Cholangiocarcinoma
Source: Cells. 2023 Jun 21;12(13):1678. doi: 10.3390/cells12131678 (PMC10340323; doi:10.3390/cells12131678)
Supplement: Supplementary file 1 [file cells-12-01678-s001.zip › cells-2386604-SM.pdf]

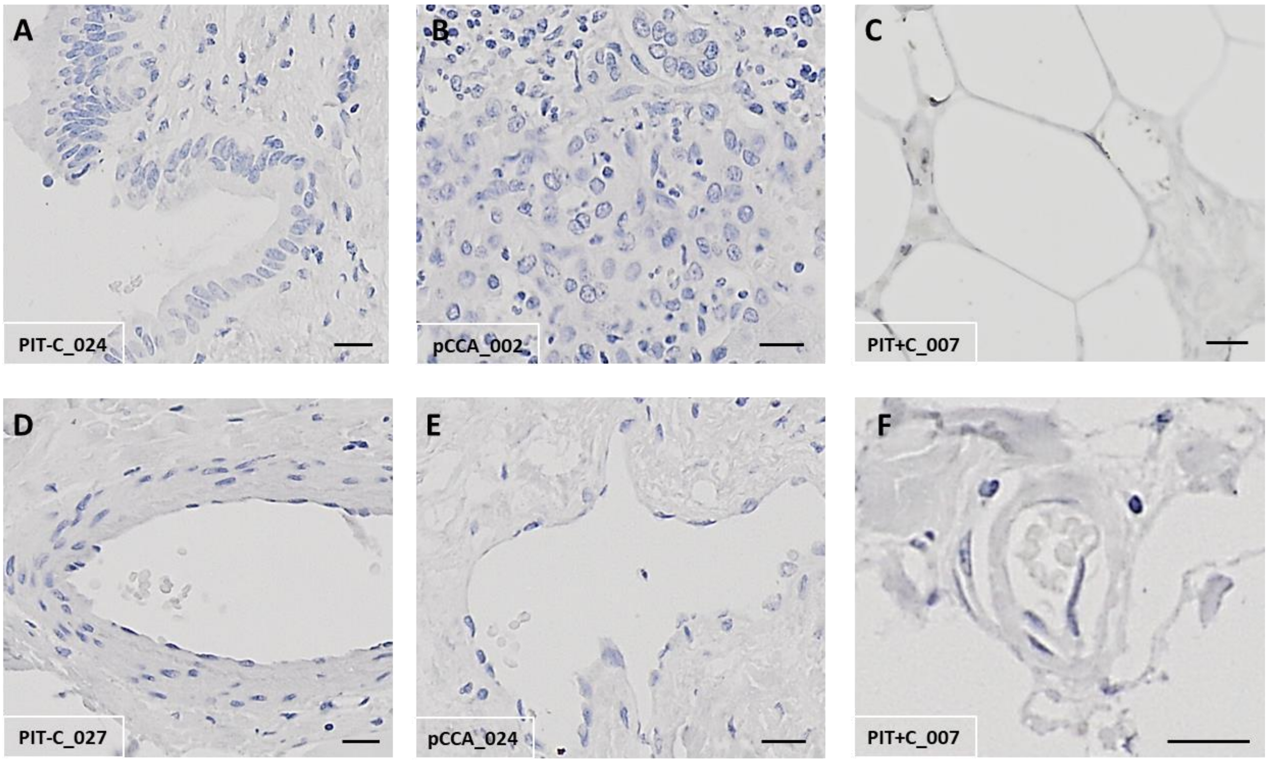

Supplement Figure S1. 2ndAb controls of representative tissues analysed by IHC. (**A**) Intact cholangiocytes of a peritumoral tissue. (**B**) tumour cells of pCCA tissue. (**C**) adipocytes, (**D**) arterial endothelial cells, (**E**) venous endothelial cells (**F**) capillar endothelial cells of peritumoral tissues. Scale bar = 20 µm.

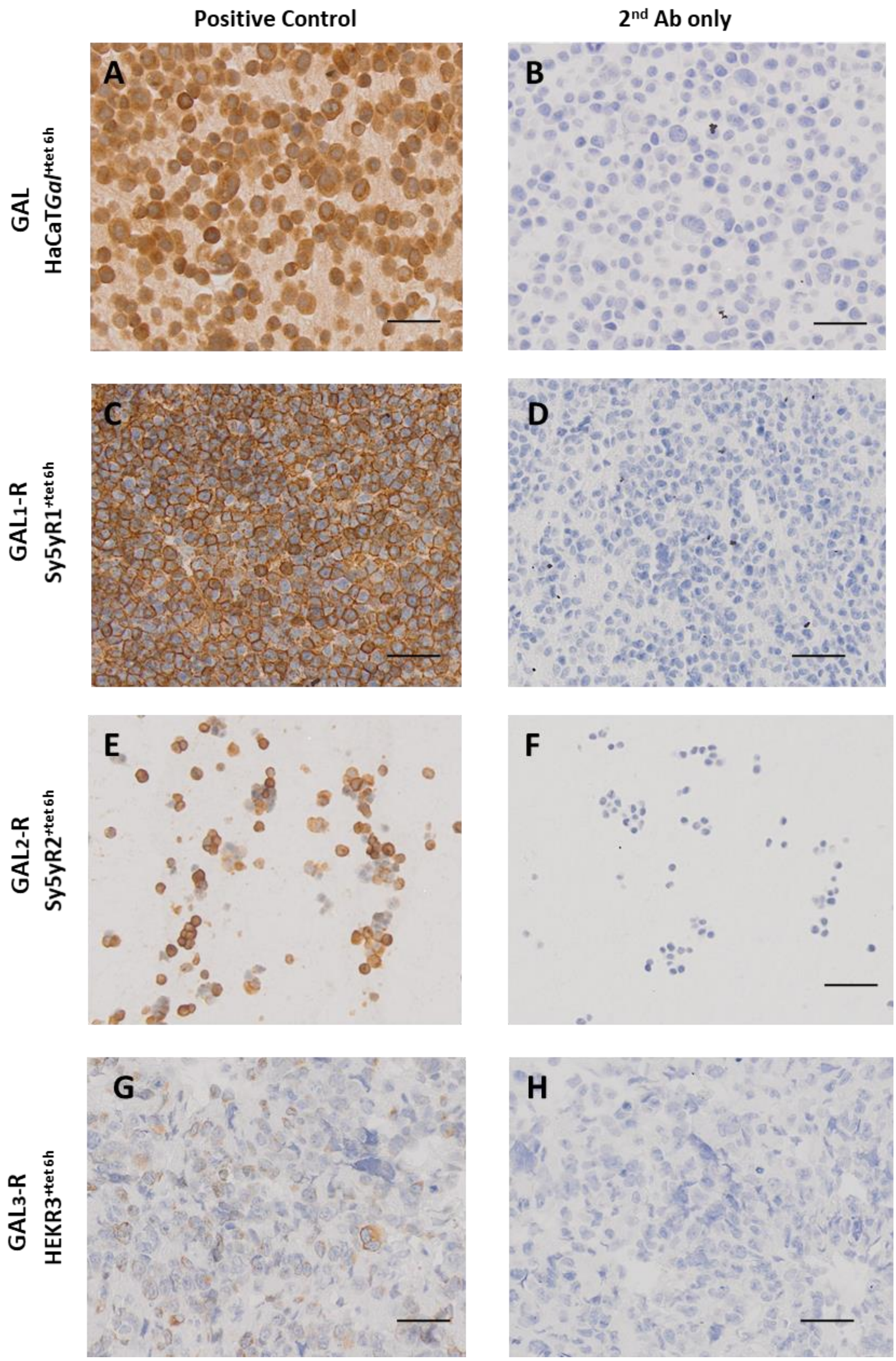

Supplement Figure S2. Positive controls and corresponding 2nd Ab controls for the GAL system included in IHC. (**A**), (**B**) GAL overexpressing HaCaTGal^{+tet6h} cells (induction of GAL expression by tetracycline treatment). (**C**), (**D**) GAL_1-R overexpressing neuroblastoma cell line Sy5yR1 (induction of GAL_1-R expression by tetracycline treatment). (**E**), (**F**) GAL_2-R overexpressing neuroblastoma cell line Sy5yR2 (induction of GAL_2-R expression by tetracycline treatment). (**G**), (**H**) GAL_3-R overexpressing human embryonic kidney cell line HEKR3 (induction of GAL_3-R expression by tetracycline treatment). Antibodies: rabbit anti-human GAL IgG, GAL_1-R IgG, GAL_2-R IgG, and GAL_3-R IgG, respectively. Antibody details are depicted in Table 1 and were validated in a previous publication of our laboratory [44]. Scale bar = 50 µm.

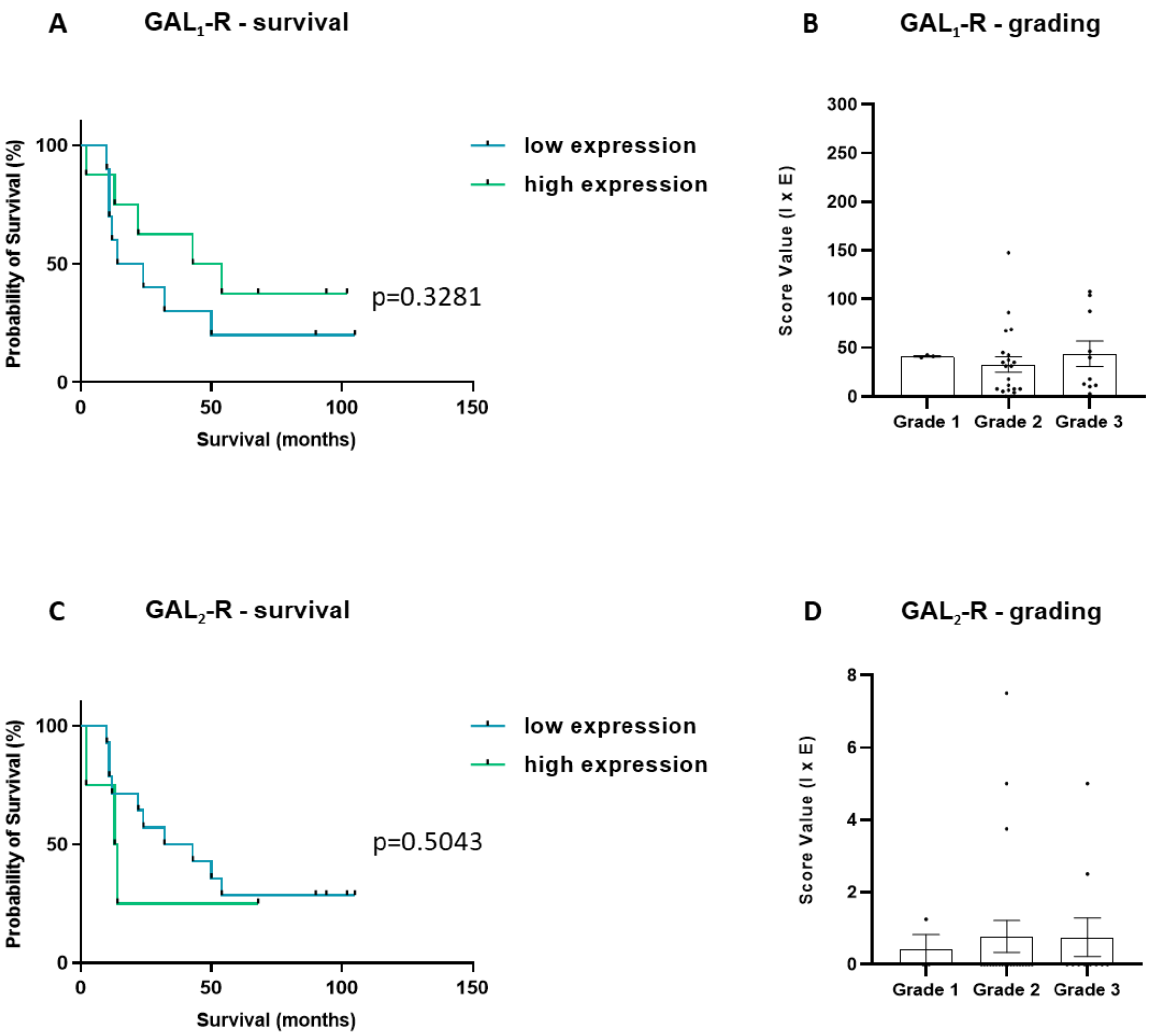

Supplement Figure S3. Kaplan–Meier curves of pCCA patients exhibiting low or high (**A**) GAL_1-R or (**B**) GAL_2-R score values in tumour cells. High expressors are shown in green and low expressors in blue. (**A**) GAL_1-R: High expressors: n=8, low expressors: n=10. (**B**) GAL_1-R expression in tumour cells of pCCA patients and associated grading (p=0.3281). (**C**) GAL_2-R: High expressors: n=4, low expressors: n=14. (**D**) GAL_2-R expression in tumour cells of pCCA patients and associated grading (p=0.5043). Grade 1: n=3, Grade 2: n=21, Grade 3: n=10. Multiple comparison tests were used for comparison, applying the Kruskal-Wallis-test and post-hoc Dunn’s multiple comparison test.

Supplementary Table S1: Clinical parameters of healthy controls and PIT

Controls (healthy)	Sex	Age [y]	Cholestasis	Cholangitis
GB01	m	87	0	0
GB02	f	58	0	0
GB03	m	79	0	0
GB04	f	47	0	0
GB05	f	82	0	0
**PIT-C**	**Sex**	**Age [y]**	**Cholestasis**	**Cholangitis**
PIT-C_002	f	43	0	0
PIT-C_005	f	53	0	0
PIT-C_009	m	74	0	0
PIT-C_010	m	67	0	0
PIT-C_013	m	72	0	0
PIT-C_015	m	72	0	0
PIT-C_023	f	66	0	0
PIT-C_024	f	82	0	0
PIT-C_027	f	74	0	0
PIT-C_029	f	62	0	0
PIT-C_030	f	48	0	0
PIT-C_033	f	75	0	0
PIT-C_041	m	75	0	0
PIT-C_042	f	67	0	0
**PIT+C**	**Sex**	**Age [y]**	**Cholestasis**	**Cholangitis**
PIT+C_006	m	78	1	0
PIT+C_007	f	71	1	0
PIT+C_017	f	75	1	0
PIT+C_020	f	90	1	0
PIT+C_022	f	68	1	0
PIT+C_026	m	74	1	0
PIT+C_028	m	72	1	0
PIT+C_036	m	81	1	0
PIT+C_038	f	69	1	0
PIT+C_040	f	68	1	0

y, years at surgery; m, male; f, female, cholestasis/cholangitis: 0-no, 1-yes;

PIT, peritumoral tissue

Supplementary Table S2: Clinical parameters of pCCA patients

Klatskin Patients	Sex	Alter [y]	Cholestasis	Cholangitis	T	N	M	V	L	Pn	R	G	Bismuth	UICC	Survival [m]	Deceased
pCCA_001	m	65	1	1	3	1	0	1	0	1	1	1	3a	3b	13	1
pCCA_002	f	43	0	1	3	0	0	0	1	1	0	2	4	2a	50	1
pCCA_003	m	74	0	1	3	0	0	0	0	1	0	2	1	2a		
pCCA_004	m	69	1	1	4	1	0	0	0	1	1	2	3a	3b		
pCCA_005	f	53	0	0	2	1	0	0	0	1	0	2	4	3c	14	1
pCCA_006	m	78	1	0	2	0					1	3	1	2		
pCCA_007	f	71	1	0	2	1	0	1		1	0	2	1	2a		
pCCA_008	f	58	1	0	2	1	0	0	1	0	0	2	3a	3b	24	1
pCCA_009	m	74	0	0	3	0	0			1	1	3	1	2a	11	1
pCCA_010	m	67	0	0	3	0	0	0	0	0	0	2	1	2a	105	0
pCCA_011	m	60	1	0	2	0	0	0	0	0	0	3	1	1b		
pCCA_012	m	64	1	0	3	1	0	0	0	0	1	2	1	3b		
pCCA_013	m	72	0	0	2	1	0	0	1	1	0	2	3a	3b	68	0
pCCA_014	m	68	1	0	3	1	0	0	1	1	0	3	1	2b	32	1
pCCA_015	m	72	0	0	2	0	0	0		1	1	1	4	2	102	0
pCCA_016	m	77	0	1	2	0	0	0	1	1	0	3	3a	2	2	1
pCCA_017	f	75	1	0	2	1	1	0	0	1	1	2	1	4	11	1
pCCA_018	f	70	1	1	3	0	0	1			1	2	1	2a		
pCCA_019	f	78	1	0	3	1		0	1	1	0	1	1	2b		
pCCA_020	f	90	1	0	3	1	0	0	1	1	0	2	1	2b		
pCCA_022	f	68	1	0	3	1	0	1	1	1	0	3	1	2b	54	1
pCCA_023	f	66	0	0	2	0	0	0	0	0	0	2	3a	2	94	0
pCCA_024	f	82	0	0	3	1	0	0	1	1	0	2	1	2b		
pCCA_026	m	74	1	0	2	1	0	0	1	1	0	2	3b	3b		
pCCA_027	f	74	0	0	2	0		0	0	2	0	2	3a	2		
pCCA_028	m	72	1	0	2	0	0	0	0	1	0	3	1	1b	90	0
pCCA_029	f	62	0	0	2	1	1	0	1	1	1	3	3b	3b	22	1
pCCA_030	f	48	0	0	2	1	0	1	0		0	3	2	3	43	1
pCCA_033	f	75	0	0	2	1		0	0	0	0	2	4	3b		
pCCA_036	m	81	1	0	2	0	0	0	0	1	0	2	2	2a	12	1
pCCA_038	f	69	1	0	3	1	0	0	0	1	1	2	3b	3b	10	1
pCCA_040	f	68	1	0	3		0				0	3	2a	3		
pCCA_041	m	75	0	0	1	0	0	1		1	0	2	1			
pCCA_042	f	67	0	0	3	1	0	1	1	1	1	2	4	4		

y, years at surgery; m, male; f, female; cholestasis/cholangitis: 0-no, 1-yes; T, tumour; N, lymph nodes; m, metastasis classification; V, invasion into venes; L, invasion into lymphatic vessels;
Pn, invasion into adjunct nerves; G, grade (1-3) of tumour; R, residual tumour after surgery; survival [m]= survival in months until day of death; deceased: 0-no, 1-yes

Supplementary Table S3: Score values of GAL expression

Controls (healthy)	Chc	Muc	Mus	Adv	Musc	LNB	SNB	Adi	ArtMuc	VenMuc	CapMuc	ArtAdv	VenAdv	CapAdv
GB01	190	51	8	8	143	40	65	233	285	223	285	130	238	238
GB02	180	59	8	0	78	25	116	5	135	135	143	155	85	185
GB03	190	35	24	10	128	176	176	170	219	238	231	225	231	231
GB04	238	19	6	3	120	145	156	29	214	190	139	190	185	185
GB05	175	6	5	6	93	80	79	123	109	176	143	68	140	208
**PIT-C**	**Chc**	**Muc**	**Mus**	**Adv**	**Musc**	**LNB**	**SNB**	**Adi**	**ArtMuc**	**VenMuc**	**CapMuc**	**ArtAdv**	**VenAdv**	**CapAdv**
PIT-C_002	65	28	0	10	4	15	6	0	4	8	4	0	6	8
PIT-C_005	238	5	4	3	14	44	40	10		178	135	50	28	
PIT-C_009	261	70	25			238	238	173	140	191	254			
PIT-C_010	231	25	33		20	115	161		110	90	208			
PIT-C_013	185		3	1	5	116	133		15	32	180	0	1	95
PIT-C_015	231	45	18	18	113	185		185			10	60	160	
PIT-C_023	219	0	4			131	113	93		118	81			
PIT-C_024	185	23	10			139	214		75	176	148			
PIT-C_029	208	6	19		40	135	190	170	85	173	130			
PIT-C_030	231	13	18			238			165	168	175			
PIT-C_033	278	44	13	70	150	170	175	225		103	98	131	175	180
PIT-C_041	285	106	70	75		231	218	231	180	190	231	195	195	238
PIT-C_042	95	0	5	1	4	124	94	93	28	34	43	48	73	85
**PIT+C**	**Chc**	**Muc**	**Mus**	**Adv**	**Musc**	**LNB**	**SNB**	**Adi**	**ArtMuc**	**VenMuc**	**CapMuc**	**ArtAdv**	**VenAdv**	**CapAdv**
PIT+C_006	166	6	5	10	30	153	48	121	21	103	73	124	5	135
PIT+C_007	160	5	9	4		128		20	0		3	0	4	30
PIT+C_017	278	3	26	5		93	93	194	53	73	120	0	73	25
PIT+C_020	225	15	20			143	149	270	88		226			
PIT+C_022	165	11	1	13	113	160	148	35	83	172	148	21	73	68
PIT+C_026														
PIT+C_028	225	13	3	8	1	219	225	90	3	145	170	11	43	219
PIT+C_036	231				65		216			175	130			
PIT+C_038	209	14	14	38	130	170	231	45	55	30	65	3	95	85
PIT+C_040	185	35	3	5	25	131	116	113			23	3	18	93

GB, gall bladder; Chc, cholangiocytes; Muc, mucosa; Mus, muscularis; Adv, adventitia; Musc, smooth muscle; LNB, large nerve bundle, SNB, small nerve bundle;
Adi, adipocytes; ArtMuc, artery in mucosa; VenMuc, vene in mucosa; CapMuc, capillary in mucosa; ArtAdv, artery in adventitia; VenAdv, vene in adventitia; CapAdv, capillary in adventitia

Supplementary Table S4: Score values of GAL$_1$-R

Controls (healthy)	Chc	Muc	Mus	Adv	Musc	LNB	SNB	Adi	ArtMuc	VenMuc	CapMuc	ArtAdv	VenAdv	CapAdv
GB01	35	0	0	41	0	4	0	3	1	1	1	8	38	48
GB02	6	0	0	0	0	0	0	0	0	0	0	0	0	0
GB03	68	14	3	1	46	170	175	19	120	119	110	150	155	154
GB04	58	0	0	0	3	68	68	13	150	38	18	0	15	0
GB05	31	0	0	0	0	0	0	0	15	15	8	0	0	0
**PIT-C**	**Chc**	**Muc**	**Mus**	**Adv**	**Musc**	**LNB**	**SNB**	**Adi**	**ArtMuc**	**VenMuc**	**CapMuc**	**ArtAdv**	**VenAdv**	**CapAdv**
PIT-C_002	118	0	28	25	0	18	6	53	0	0	0	3	5	0
PIT-C_005	0	0	0	0	0	0	0	45		8		10	3	
PIT-C_009	85	0	0			93	26	79	3	35	56			
PIT-C_010	19	0	0		0	0	0	0	0	0	0			
PIT-C_013	135	0	0	18	0	166	105		0	28	0	3	3	34
PIT-C_015	43	0	0	0	0	38	3	9	0	45	0	0	0	0
PIT-C_023	131	0	0			166	0	179		128	100			
PIT-C_024	88	0	0	0	33	48	0		70	88	0			
PIT-C_027	18	0	0		0	78	9	3	10	88	18			
PIT-C_029	30	0	0		0	46	43	0	36	40	0			
PIT-C_030	151	43	40				139		5	93	88			
PIT-C_033	128	50	24	25	0	225	238	18		0	0	5	0	38
PIT-C_041	53	0	0	11		29	21	8	6	5	0	8	9	23
PIT-C_042	18	0	0	43	0	0	0	35	1	0	0	0	0	0
**PIT+C**	**Chc**	**Muc**	**Mus**	**Adv**	**Musc**	**LNB**	**SNB**	**Adi**	**ArtMuc**	**VenMuc**	**CapMuc**	**ArtAdv**	**VenAdv**	**CapAdv**
PIT+C_006	0	0	0	1	0	9	0	15	0	0	0	0	0	0
PIT+C_007	239	15		23		105	80	180	95		155	194	43	
PIT+C_017	80	13	10	106		39	80	40	101	101	68	9	170	45
PIT+C_020	11	0	0			63	10		3		0			
PIT+C_022	113	0	0	38	18	0	113	40	38	80	63	8	8	0
PIT+C_026														
PIT+C_028	163	5	1	28		41	40	38	8	53	18	30	95	0
PIT+C_036	18	0	5		11		53			29	18			
PIT+C_038	89	0	0	11	0	29	28	10	8	0	30	0	0	35
PIT+C_040	8	0	0	13	0	0	0	13			0	0	0	0

GB, gall bladder; Chc, cholangiocytes; Muc, mucosa; Mus, muscularis; Adv, adventitia; Musc, smooth muscle; LNB, large nerve bundle, SNB, small nerve bundle;

Adi, adipocytes; ArtMuc, artery in mucosa; VenMuc, vene in mucosa; CapMuc, capillary in mucosa; ArtAdv, artery in adventitia; VenAdv, vene in adventitia; CapAdv, capillary in adventitia

Supplementary Table S5: Score values of GAL_2-R

Controls (healthy)	Chc	Muc	Mus	Adv	Musc	LNB	SNB	Adi	ArtMuc	VenMuc	CapMuc	ArtAdv	VenAdv	CapAdv
GB01	4	0	0	3	0	0	0	0	0	0	0	0	0	0
GB02	0	5	0	0	0	0	0	0	0	0	8	0	5	0
GB03	0	0	0	0	0	0	0	0	0	3	0	0	0	0
GB04	5	3	0	0	0	0	0	0	0	0	0	0	0	0
GB05	0	5	0	5	0	0	0	1	0	0	0	0	0	0
**PIT-C**	**Chc**	**Muc**	**Mus**	**Adv**	**Musc**	**LNB**	**SNB**	**Adi**	**ArtMuc**	**VenMuc**	**CapMuc**	**ArtAdv**	**VenAdv**	**CapAdv**
PIT-C_002	5	23	5	8	1	0	0	10	3	4	3	1	1	1
PIT-C_005	3	0	0		0	0	0	1	0	0	0			
PIT-C_009	0	0	0			0	0	0	0	0	0			
PIT-C_010	0	0	0		0	0	0		0	0	0			
PIT-C_013	0	0	0	0	0	0	0		0	0	0	0	0	0
PIT-C_015	0	0	0	0	0	0	0	5	0	0	0	0	0	0
PIT-C_023	0	0	0	0		0	0	0	0	0	0	0	0	0
PIT-C_024	0	0	0			0	0		0	0	0			
PIT-C_027	0	0	0		0	0	0	0	0	0	0			
PIT-C_029	3	0	0		0	0	0	0	0	0	0			
PIT-C_030	0	0	0			0	0		0	0	0			
PIT-C_033	0	0	0	0	0	0	0	0	0	0	0	0	0	0
PIT-C_041	0	0	0	0		0	0	5	0	0	0	0	0	0
PIT-C_042	8	0	1	35	0	0	0	15	1	3	1	4	1	0
**PIT+C**	**Chc**	**Muc**	**Mus**	**Adv**	**Musc**	**LNB**	**SNB**	**Adi**	**ArtMuc**	**VenMuc**	**CapMuc**	**ArtAdv**	**VenAdv**	**CapAdv**
PIT+C_006	3	13	1	5	0	0	0	4	0	0	0	0		3
PIT+C_007	0	1	1	3				0	0	0	0	0	0	0
PIT+C_017	0	0	0	5		0	0	0	0	0	0	3	0	0
PIT+C_020	0	0	0			0	0	0	0	0	0	0		
PIT+C_022	0	0	0	0	0	5	0	0	0	0	0	0	0	0
PIT+C_026						0	0	0						
PIT+C_028	0	0	0	0	0	0	0	0	0	0	0	0	0	0
PIT+C_036	0	0	0		0		0				0			
PIT+C_038	0	0	0	0	6	0	0	0	0	0	0	0	0	0
PIT+C_040	0	0	23	4	0	3	0	10			0			1

GB, gall bladder; Chc, cholangiocytes; Muc, mucosa; Mus, muscularis; Adv, adventitia; Musc, smooth muscle; LNB, large nerve bundle, SNB, small nerve bundle;

Adi, adipocytes; ArtMuc, artery in mucosa; VenMuc, vene in mucosa; CapMuc, capillary in mucosa; ArtAdv, artery in adventitia; VenAdv, vene in adventitia; CapAdv, capillary in adventitia

Supplementary Table S6: Score values of GAL_3-R

Controls (healthy)	Chc	Muc	Mus	Adv	Musc	LNB	SNB	Adi	ArtMuc	VenMuc	CapMuc	ArtAdv	VenAdv	CapAdv
GB01	13	0	0	0	0	0	0	0	0	0	155	0	3	0
GB02	30	0	0	0	0	0	0	0	0	0	19	0	0	0
GB03	55	0	0	0	0	0	0	0	0	0	26	0	5	0
GB04	19	0	0	0	0	0	0	0	0	0	150	0	0	0
GB05	0	0	0	0	0	0	0	0	0	0	115	0	0	0
**PIT-C**	**Chc**	**Muc**	**Mus**	**Adv**	**Musc**	**LNB**	**SNB**	**Adi**	**ArtMuc**	**VenMuc**	**CapMuc**	**ArtAdv**	**VenAdv**	**CapAdv**
PIT-C_002	0	0	0	0	0	0	0	0	0	0	0	0	0	0
PIT-C_005	18	0	0	0	0	0	0	0	0	0	0	0	0	0
PIT-C_009	4	0	0			0	0	0	0	0	0			
PIT-C_010	0	0	0		0	0	0		0	0	0			
PIT-C_013	8	0	0	0	0	0	0	0	0	0	0	0	0	0
PIT-C_015	0	0	0	0	0	0	0	0	0	0	0	0	0	0
PIT-C_023	75	0	0	0	0	0	0	3	0	0	170	3	0	0
PIT-C_024	0	0	0		0	0	0		0	0	0			
PIT-C_027	0	3	0		0	0	0	0	0	0	0			
PIT-C_029	3	0	0		0	0	0	0	0	0	15			
PIT-C_030	5	0	3			0	0	0	0	0	73			
PIT-C_033	0	0	0	0		0	0	0	0	0	0	0	0	0
PIT-C_041	3	0	0	0	0	0	0	0	0	0	5	0	0	0
PIT-C_042	4	0	0	0	0	0	0	0	0	0	0	0	0	0
**PIT+C**	**Chc**	**Muc**	**Mus**	**Adv**	**Musc**	**LNB**	**SNB**	**Adi**	**ArtMuc**	**VenMuc**	**CapMuc**	**ArtAdv**	**VenAdv**	**CapAdv**
PIT+C_006	10	0	0	0	0	0	0	0	0	0	0	0	0	0
PIT+C_007														
PIT+C_017	140	5	0	0	0	0	0	13	0	0	219	0	0	25
PIT+C_020	0	0	0	0	0	0	0	0	0		0	0	0	0
PIT+C_022	18	0	0	0	0	0	0	0	0	0	20	0	0	3
PIT+C_026														
PIT+C_028	0	0	0	0	0	0	0	0	0	0	0	0	0	0
PIT+C_036	0	0	0	0	0	0	0	0		0	0			
PIT+C_038	0	0	0	0	0	0	0	0	0	0	0	0	0	0
PIT+C_040	15	0	0	0	0	0	0	0	0	0	0	0	0	0

GB, gall bladder; Chc, cholangiocytes; Muc, mucosa; Mus, muscularis; Adv, adventitia; Musc, smooth muscle; LNB, large nerve bundle, SNB, small nerve bundle;

Adi, adipocytes; ArtMuc, artery in mucosa; VenMuc, vene in mucosa; CapMuc, capillary in mucosa; ArtAdv, artery in adventitia; VenAdv, vene in adventitia; CapAdv, capillary in adventitia

Supplementary Table S7: Score values of GAL and GAL_{1-3}-R in pCCA patients

pCCA Patient	GAL	GAL_1-R	GAL_2-R	GAL_3-R
pCCA_001	116	40	1	0
pCCA_002	238	8	0	0
pCCA_003	135	0	0	0
pCCA_004	143	31	0	0
pCCA_005	139	0	8	8
pCCA_006	46	13	0	1
pCCA_007	143	8	0	0
pCCA_008	208	35	0	0
pCCA_009	166	11	0	0
pCCA_010	238	35	0	0
pCCA_011	118	104	0	0
pCCA_012	231	148	0	0
pCCA_013	238	43	4	0
pCCA_014	95	18	0	0
pCCA_015	285	41	0	0
pCCA_016	161	46	3	3
pCCA_017	128	6	0	5
pCCA_018	143	45	0	0
pCCA_019	238	43	0	0
pCCA_020	254	8	0	0
pCCA_022	175	40	0	0
pCCA_023	238	69	0	0
pCCA_024	190	11	0	1
pCCA_026	238	68	0	0
pCCA_027	238	4	0	0
pCCA_028	190	3	0	0
pCCA_029	88	108	0	0
pCCA_030	190	88	0	9
pCCA_033	285	86	0	8
pCCA_036	238	18	0	36
pCCA_038	175	31	0	81
pCCA_040	143	10	5	0
pCCA_041		5	5	15
pCCA_042	83	38	0	0
